# Southern Spitsbergen coastal permafrost - repeated seismic survey supported by GPR

**DOI:** 10.1038/s41597-025-06327-4

**Published:** 2025-11-21

**Authors:** Mariusz Majdański, Artur Marciniak

**Affiliations:** 1 Institute of Geophysics, Polish Academy of Sciences, Warsaw, Poland; 2 Department of Geodesy and Geoinformation, TU Wien, Vienna, Austria

**Keywords:** Environmental impact, Cryospheric science, Geophysics

## Abstract

We present a 2D seismic and GPR data set collected to study seasonal dynamics of coastal Permafrost in the high Arctic. The data set was recorded as three repeated surveys in 2017 and 2018, aiming to image the extreme stages of the coastal Permafrost active layer. High resolution active source seismic, gathered in September 2017, April & May 2018, allows both near-surface analysis and reflection imaging of deep geological structures. Supporting GPR data, recorded in April, August and September 2018, gives high-resolution insight into the dynamically changing Permafrost’s active layer. Both data sets are provided in standard formats with additional survey geometry information in external files.

## Background & Summary

Near-surface geophysics methods, including active seismic and ground-penetrating radar (GPR), allow precise shallow subsurface imaging. Those methods differ in physical principles, resulting in various resolutions and penetration depths. Their combination allows precise structural analysis, and once repeated, shows changes in the structure, which allows tracking environmental processes on their scale and dynamics.

Permafrost is important in high-latitude and high-altitude ecosystems, covering about 11% of exposed Earth’s land^1^. The state of permafrost and how it is changing are key signs of environmental shifts happening in the Arctic. In Svalbard, almost all land not covered by glaciers has permafrost beneath it. Changes in climate such as air temperature, snow depth, and longer periods of warm weather, are causing the permafrost to warm up and the active layer (the top part that thaws each summer) to get deeper^2^.

In southwest Spitsbergen, Svalbard, the temperature changes we have seen over the last 40 years (from 1979 to 2018) are way bigger than the global average^3^. When permafrost melts or degrades, it changes the ground’s properties, affecting water flow, soil stability, and ecosystem processes. The active layer used to be about 0.2 meters deep and has grown to around one meter over the last four decades; with the Arctic warming even faster due to polar amplification, it is expected to get deeper still^4^.

Geophysical tools cannot get the exact temperature of what is underground, but they can indirectly tell us about changes in the ground’s physical properties. Laboratory tests show that when a rock is above 0 °C, especially those with low porosity, their seismic P-wave speed increases by about 11% compared to when it is colder^5^. This change is enough to spot permafrost in a single rock^6^. In areas where rock types and their distribution do not change much, we can detect the edge of permafrost by measuring these seismic changes, even at pretty deep levels. When high-porosity rocks freeze, their P-wave speed can double^7^.

Recently, scientists have been studying permafrost in mountain and polar regions using different geophysical methods. These include satellite-based gravity measurements^8^, electrical resistivity tomography (ERT)^9^, surface wave analysis^10^, travel time tomography^11^, shear wave^12^, and seismic reflection imaging^13^. However, there is little information on how seasonal changes in the active layer affect seismic waves. Most studies have been done during the summer melt season. An example of reflection imaging with data from two difference seasons, that are part of discussed dataset, shows how significant those variations are^14^. Other example, seismic work on Svalbard’s Adventdalen has helped image deep underground structures, like where to store CO2 safely^15^. These studies show we can get detailed images of the subsurface down to about 1 km depth if shallow active layers seasonal variation are included in data processing.

**Fig. 1:**
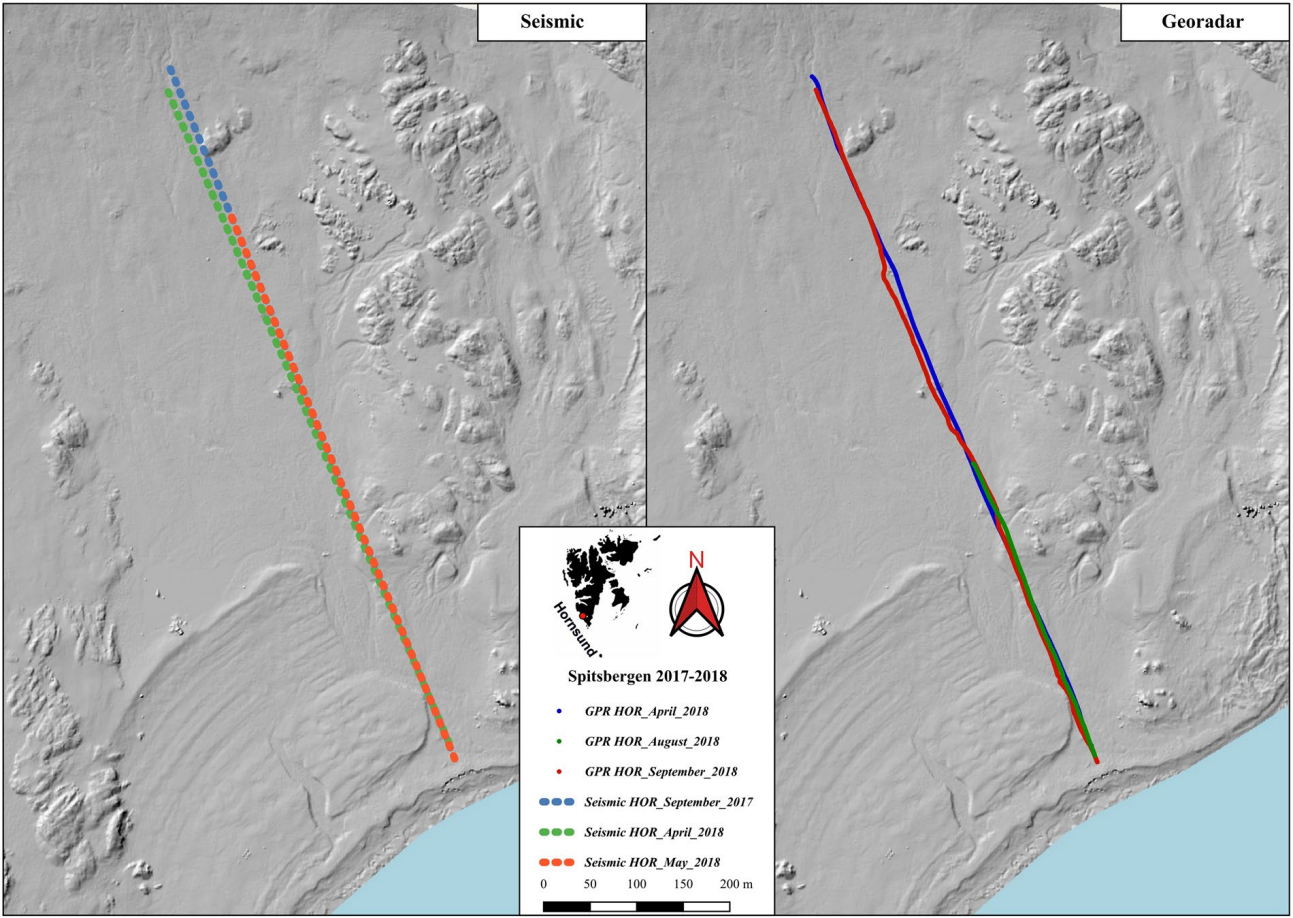
Digital elevation map of Fuglebekken catchment with marked seismic and GPR profiles. Central inlet shows the location of the site in the Hornsund, the south of Spitsbergen. HOR is abbreviation for Hornsund survey.

The study area is situated on the southern shore of Hornsund Fjord in southwestern Spitsbergen (Fig. 1), within the Fuglebekken basin. The region is characterised by geomorphological and ecological diversity, as evidenced by the southern mountain slopes of Ariekammen-Fugleberget and the Fuglebergsletta coastal plain^16^. Elevated marine terraces support a variety of tundra vegetation, while the Fuglebergsletta plain is predominantly covered by marine gravel. The mountain slopes are characterised by sediments of washed-out debris, solifluction tongues, rock streams, alluvial cones, and exposed bedrock.

Geologically, the area is underlain by Cambrian and Ordovician sedimentary successions, comprising sands, gravels, and clays overlaying Precambrian crystalline bedrock. The crystalline basement, part of the Lower and Middle Hecla Hoek geological formations, includes metamorphic quartzites, schists, paragneisses, marbles, and amphibolites. Tectonic activity, glacial abrasion, and frost weathering during the transition from the Pleistocene to the Holocene have resulted in extensive fracturing of these rocks in the near-surface zone. Marine terraces play a crucial role in the region’s geological framework. These abrasive platforms, distinguished by age-specific marine sediments, were formed through isostatic uplift^16^. Based on previous studies, three principal terrace levels have been identified^17^. The uplift of these terraces was spatially irregular, influenced by factors such as local tectonic activity^18^, the distribution of former ice-sheet loads, and deglaciation history^19^. These platforms have undergone significant modification due to erosion, colluvial accumulation, and solifluction processes on slopes^20^. Additionally, glacial and fluvioglacial landforms—including moraines and deposits of soil and rock—are superimposed on the uplifted marine terraces in the foreland of the Hans Glacier. These features have been subject to rapid changes within the periglacial environment. The maximum extent of the Hans Glacier was marked by the end moraine deposited around 1900, coinciding with the end of the Little Ice Age^21^. This end moraine is characterised by a prominent accumulation of eroded moraine sediments with a buried ice core. Blocks of dead ice are also present within the ground moraine sediments, resulting from the glacier’s advance and retreat. The degrading dead ground moraine zone extends from the current glacier front to the aforementioned terminal moraine^16^. Concurrently, a network of smaller lateral moraines has developed around these ground moraine formations.

The presented data set (Fig. 1) allows studying temporal changes and dynamics of the permafrost’s active layer^12^. It also allows analysis of deeper structures and hydrogeological processes affecting them^14^.

## Methods

**Fig. 2:**
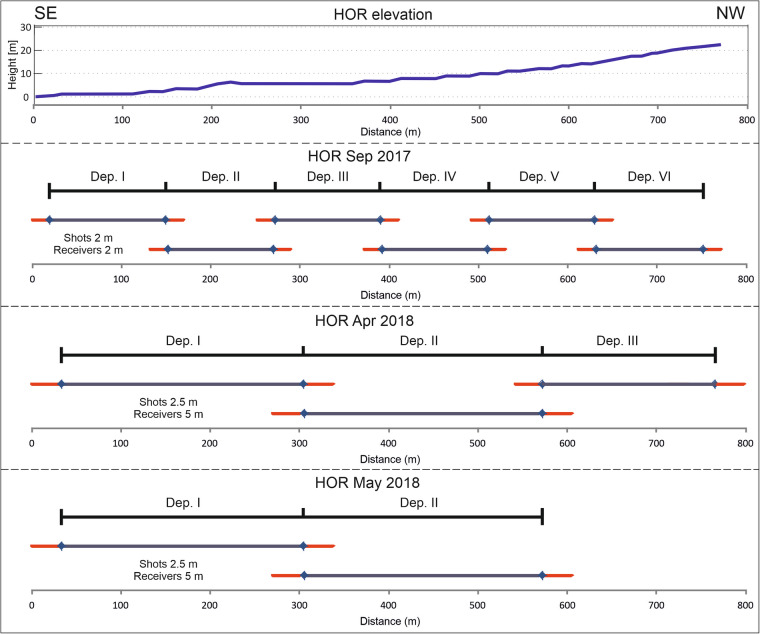
Schematic survey geometry used for three seismic profiles. The top panel shows changing elevation. Other panels show deployments in September 2017, April 2018 and May 2018 with marked extended shot positions against station spread. HOR – name of Hornsund survey; Dep. – deployment.

**Fig. 3:**
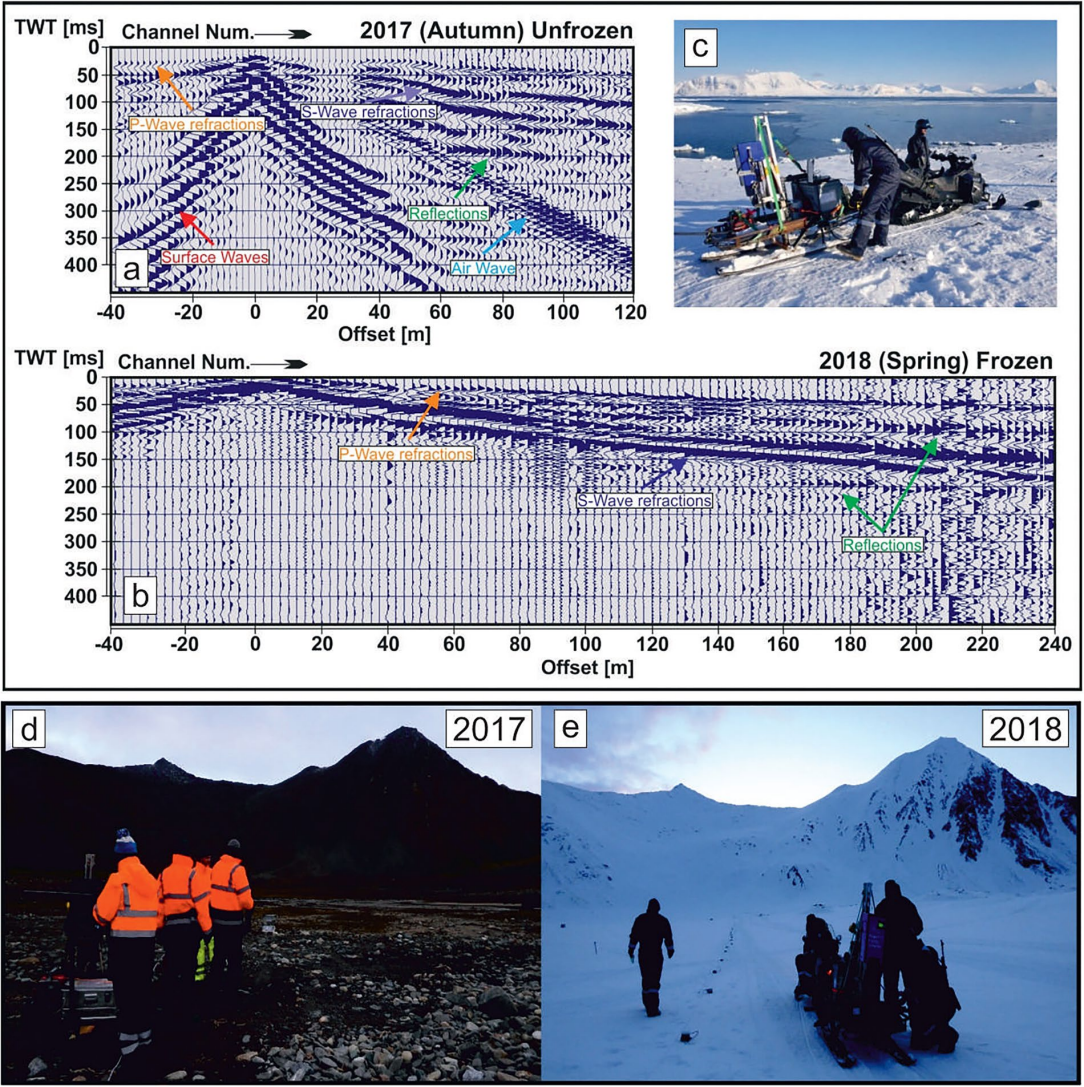
Example wavefield and field conditions in 2017 and 2018 surveys. Panels (**a,b**) show differing wavefields, without surface waves in 2018. (**c**) PEG-40 seismic source mounted on sledges. Panels (**d**) and (**e**) show different field conditions in 2017 and 2018.

The seismic surveys were designed to image the change of frozen ground and the unfrozen active layer of permafrost along profile from the sea shore to base of the mountain. Two fieldwork campaigns were conducted in two seasons: maximum thaw on 21–28 September 2017 and maximum snow cover and frozen ground from April 15 to May 10 2018. The profiles were positioned using GPS systems (manual and built-in receivers in seismic stations) and measuring tapes and markers. Classical differential GPS was not operational in these conditions in 2017–2018 due to a lack of a nearby reference base station. Seismic signals were recorded by standalone recorders (DATA-CUBE by Digos) with 1 C 4.5 Hz geophones, recording with 400 Hz sampling. We used 60 stations in multiple deployments (see Fig. 2). Receiver spread varied from 2 m in 2017 to 5 m in 2018. In frozen conditions, we could observe clear signals for larger offsets, so the profile length was extended to 350 m with 5 m spacing for each deployment. We used PEG-40 accelerated weight-drop supported with a GPS-based timing system as a seismic source. The source was mounted on a wheeled cart in unfrozen conditions and on a sledge in show conditions (see Fig. 3). In both seasons, the source was hitting a metal plate lying on the ground or hard snow/ice. To maximise signal to noise ratio, we used three shots in each position, and up to 6 shots during the snow season, which were vertically stacked. In snow conditions, the first strike compacts the snow, so the time is significantly different from consecutive shots. Because of that, the first strikes were discarded from the vertical stack. Shot spacing was 2 m in 2017 and 2.5 m in 2018. To improve fold overlapping shots were used when possible.

**Fig. 4:**
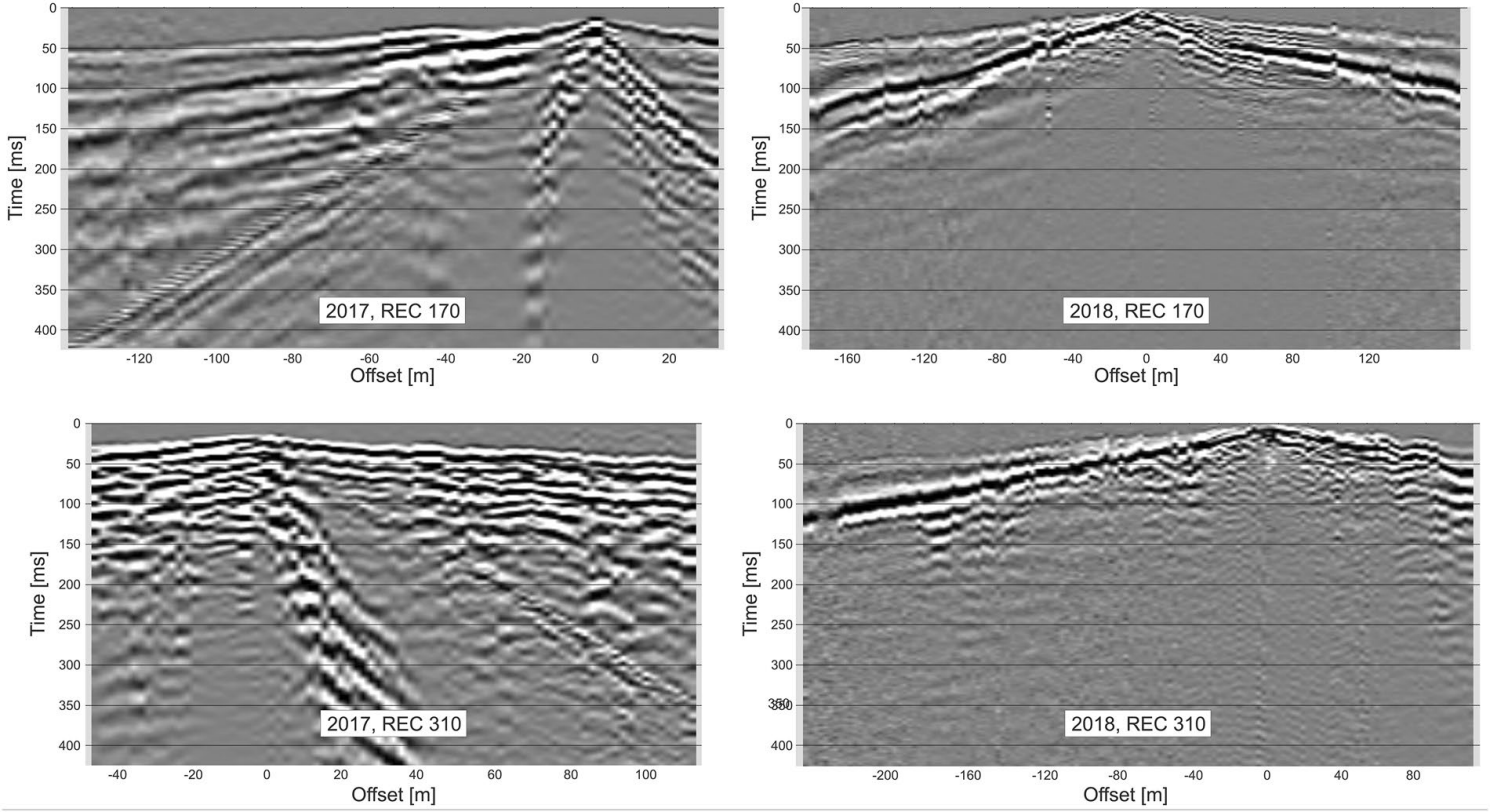
Examples of seismic gathers for receiver 170 and 310 for September 2017 survey (left) and April 2018 survey (right). Visualised with interpolation of balanced traces after filtering (8–160 Hz). Both the first arrivals and the complexity of the wavefield are significantly different.

To ensure data quality, careful QC and pre-processing of seismic data were applied, including removal of bad and noisy recordings. Continuous recording by DATA-CUBEs was cut according to measured shot times. To process the data we used the Globe Claritas program which uses SEGY format for seismic data. After initial QC, the data were vertically stacked, and then a final pass on control was made to eliminate bad datum (Fig. 4). Survey geometry data were added to SEGY headers, including source and receiver number, their position in meters, CDP, and offset (see Table 1). Multiple deployments were sorted into a single SEGY file for each survey, making them ready for the main processing and imaging stages.

**Table 1:** The header information stored in the SEG-Y files for seismic data.

Header	Start byte	End byte
Trace number	5	8
Shot number	9	12
Receiver X [m]	81	84
Shot X [m]	73	76
CDP [m]	21	24
offset [m]	37	40

The GPR data was gathered in 2018 using a Mala 30 MHz unshielded Rough terrain antenna. Such a configuration is optimal for the visualisation of structures down to 25–40 m in Polar conditions, which is the blind spot of seismic reflection techniques. The trace sampling was set to 0.9 m in April, and 0.8 m in August and September. Such data density proved optimal for the given profile length and depth of the surveyed targets.

**Fig. 5:**
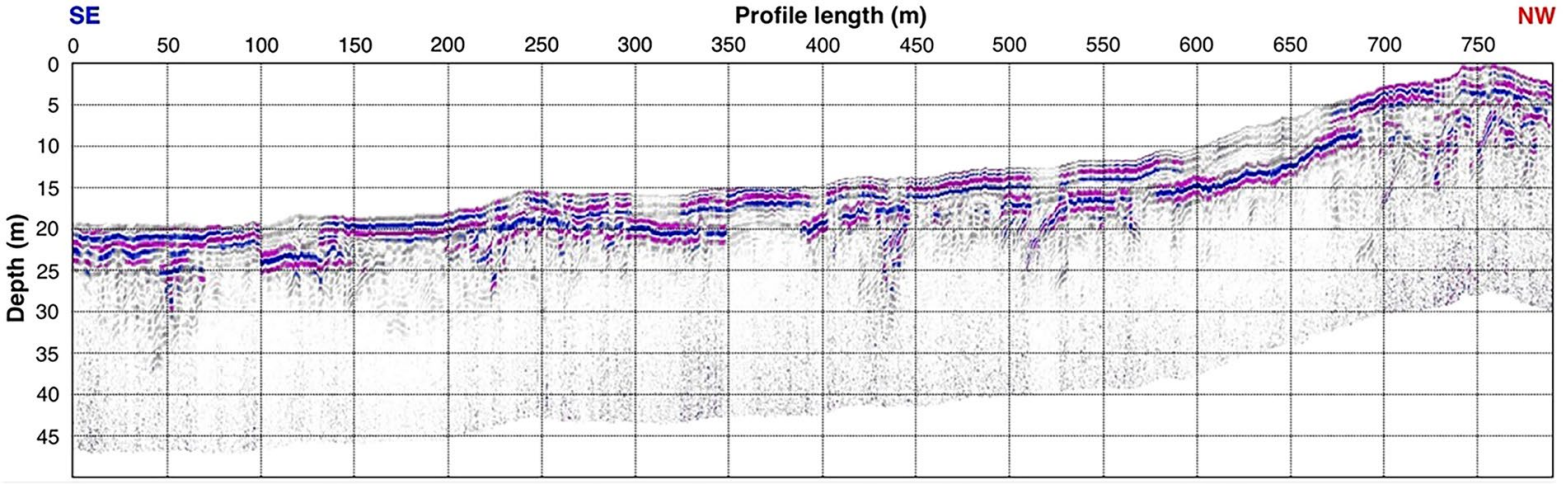
GPR Sep 2018 data showing strong reflectivity related to the surface and bottom of the permafrost’s active layer.

The example processing of the data from September 2018 (Fig. 5) consisted of data normalisation, bandpass frequency filtering (10–80 MHz range), moving start time to correct position to remove air electromagnetic wave traveltime, background noise removal, linear and exponential signal gain, time cut at 500 ns, time to depth conversion assuming averaged 11 cm/ns wave speed and finally topographic correction to add elevation shifts.

The data revealed key structures, including water-saturated zones, as well as the thickness of the active layer. Moreover, multiple deeper reflections can be noticed, linked to the marine terrace formation of the studied area.

## Data Records

The data described here are available in the dataportal repository by the Institute of Geophysics, Polish Academy of Sciences^22^. They contain three seismic data sets in standard SEGY format (Rev. 0, little endian, IEEE floating point format, 3200-bit textual header) for September 2017, April 2018, and May 2018. Seismic data have 400 Hz time sampling and 1 s record time. Additionally, three text files with the same names as survey geometry are available. In a separate directory, GPR data are available in subfolders for April, August and September 2018 measurements. Data are in standard MALA RD3 format, including geometry and supplementary information in txt files (4 files: .cor, .rad, .mrk, .rd3) for each measurement. The recording time is ~3 s. for each GPR dataset. The GPS data from the internal recorder is stored in a .cor file, including additional time marks for each separate trace, and spacing between them.

## Technical Validation

Quality of seismic data. Provided seismic data with headers in SEGY format are sorted to the shot domain, but can be easily sorted to the receiver domain for quality check. This procedure allows verification of correct geometry, timing and removal of wrong traces, and this was done before publishing this dataset. Those files were tested using different commercial and open-source programs: Globe Claritas (Petrosys), Seisee 2.22, ReflexW 10.3 (Sandmaier Geophysical Research).

Quality of GPR data. Data quality was described in previous analysis, where part of dataset was used for near-surface correction of seismic data^13^. GPR data in MALA RD3 format were tested using ReflexW 10.3 software.

## Usage Notes

The data are open and meet FAIR principles. They can be downloaded without logging in or permission from IG PAS Dataportal^22^. Part of these data were used for travel time tomography, surface wave dispersion analysis (MASW), reflection imaging, but could be useful for other analyses.

## Data Availability

All data described in this paper are open with licence CC BY 4.0 and available in the dataportal of the Institute of Geophysics, Polish Academy of Sciences with the link. https://dataportal.igf.edu.pl/dataset/repeated-active-seismic-and-gpr-data-for-coastal-permafrost-in-hornsund and marked with dataset DOI number 10.25171/InstGeoph_PAS_IGData_87656744 as described by Majdański^22^. Data contains three active seismic and GPR files. Seismic data are provided in standard SEGY format used by both industry and academic community. Seismic data contains three SEGY files for September 2017, April 2018, and May 2018 surveys. Additional text files with corresponding names describe seismic survey geometry that is also included in standard SEGY headers. GPR data are provided in standard widely used MALA RD3 format, and are available in three subfolders for April, August and September 2018 surveys.

## Code availability

No custom code has been used to generate this dataset. Standard commercial and open-source software can open and process it.
